# Completion total pancreatectomy (CTP) for isolated PDAC recurrence

**DOI:** 10.6026/973206300223165

**Published:** 2026-05-31

**Authors:** Praveen Singh Baghel, Shailendra Singh Nargesh, Yogesh Savaner, Shivam Singh Tomar, Pinky K.M, Prashant Chauhan

**Affiliations:** 1 Departments of General Surgery, Dr Laxmi Narayan Pandey Medical College, Ratlam, Madhya Pradesh, India

**Keywords:** Completion total pancreatectomy (CTP), pancreatic ductal adenocarcinoma (PDAC), isolated recurrence, disease-free interval, overall survival, salvage surgery

## Abstract

Pancreatic ductal adenocarcinoma (PDAC) is characterized by a poor prognosis and currently represents the fourth leading cause of cancer-related death. Outcomes of completion total pancreatectomy (CTP) for recurrent pancreatic ductal adenocarcinoma (PDAC) remain insufficiently defined. Therefore, it is of interest to evaluate perioperative safety and oncological outcomes in 84 patients undergoing CTP for isolated PDAC recurrence. Clinical variables including (R0) resection rate, operative parameters, morbidity, mortality and survival outcomes were analyzed. CTP achieved R0 resection in most cases with acceptable morbidity and mortality, while improved survival was associated with disease-free interval >24 months, CA 19-9 <37 U/mL and absence of lymph node metastasis. Thus, we show CTP as a viable salvage option providing meaningful long-term survival in selected patients with recurrent PDAC.

## Background:

Pancreatic ductal adenocarcinoma is a disease that is characterized by silent onset and early spread to other systems [1]. Surgical operation, whether by pancreatoduodenectomy or distal pancreatectomy is the only effective means of curative treatment in localized cases [2]. After intensive operative procedures and the modern adjuvant therapy, most patients ultimately develop some type of disease relapse [3]. Although distant metastasis is the most widespread failure mode, in a small proportion of patients, isolated recurrence in the remaining pancreas is observed [4]. A major diagnostic challenge is to differentiate between a recurrence at a late stage and a metachronous primary tumor in the remaining tissue [5]. Traditionally, clinicians considered the recurrence of the remnant as an indication of systemic failure, most often choosing palliative chemotherapy instead of additional surgery [6]. Recently, completion total pancreatectomy has been gaining popularity as a definitive salvage procedure to obtain a secondary R0 resection margin [7]. A completion pancreatectomy is technically torturous because the surgeons have to pass through thick fibrosis and deformed structures due to the previous surgery [8]. The excision of the whole organ leads to an immediate and definite apancreatic condition, which implies the lifelong hormone replacement and enzyme replacement [1]. The resulting Type 3c diabetes is especially unstable as the cells that produce glucagon are destroyed leading to glucose control that is extremely hard [3]. Existing data indicates that survival advantages of surgical salvage can balance the metabolic hazards when patients are properly screened [2]. It is important to identify certain biomarkers and clinical signs to avoid carrying out morbid surgeries on patients with occult systemic disease [5]. The length of the disease-free period, CA 19-9 trends, among other factors, are also becoming relied upon in order to measure the biology of the recurrence [9]. CTP is not only technically feasible but also oncologically beneficial, with acceptable perioperative safety and survival outcomes. Therefore, it is of interest to assess the perioperative safety and long-term oncologic outcomes in this study in order to determine who actually benefits by completion pancreatectomy.

## Materials and Methods:

This retrospective longitudinal study cohort would be identified by a cross-referenced query of an institutionally maintained prospectively maintained surgical database between January 2024 and December 2025. Only patients with isolated pancreatic ductal adenocarcinoma (PDAC) recurrence confined to the pancreatic remnant after an initial curative-intent excision were allowed to participate. To maintain an oncologic homogeneity of the study group, strict exclusion protocol was developed in relation to any patients showing simultaneous systemic dissemination, such as occult peritoneal carcinomatosis or hepatic metastases detected in the pre-operative staging. Isolated recurrence was confirmed by high-resolution multidetector computed tomography (MDCT) and during the second half of the study, by using integrated F-FDG positron emission tomography/computed tomography (PET/CT) to increase the sensitivity of metastatic detection. A formal assessment by a multidisciplinary tumor board was done on all applicants to completion total pancreatectomy (CTP) to guarantee technical resect ability and physiologic fitness. The operation plan focused on the secondary R0 resection and required careful dissection of the retroperitoneal area and frequently, the removal of dense fibrotic tissue that comes along with the first operation. The operative strategy that was employ depending on the anatomy of the original resection (pancreatoduodenectomy versus distal pancreatectomy), was either formal head resection, or mobilization of the remaining pancreatic tail and vascular reconstruction where tumor encasement was anticipated. Standardization of post-operative care followed an Enhanced Recovery after Surgery (ERAS) paradigm, with a particular emphasis upon early intensive insulin therapy and weight-adjusted pancreatic enzyme replacement therapy (PERT) to counterbalance the metabolic impact of the pancreatic condition.

## Statistical analysis:

The oncologic outcome was based on five-year overall survival (OS) as the time interval between the date of completion of pancreatectomy and the date of death or the date of last clinical follow-up. Continuous variables were reported as median with interquartile range (IQR) and compared with the help of the while the categorical data were evaluated by Chi-square or Fisher exact test as needed. The Kaplan-Meier method was used to estimate survival probabilities.

## Results:

The ultimate examination included 84 patients who have gone through completion total pancreatectomy (CTP) because of isolated recurrence. The age of the cohort at the time of the secondary operation was 66.0 (IQR 58-72) and median disease-free interval (DFI) since the first operation was 28.4 months, as shown in Table 1. Out of the 84 patients, 62% (n = 52) patients had a history of pancreatoduodenectomy and the rest 38% (n = 32) had a history of distal pancreatectomy. All cases were found to have invasive ductal adenocarcinoma pathologically examined in recurrent lesions, with a median tumor size of 2.2 cm and an R0 resection rate of 82.1%. The mean length of time to perform CTP was 345 minutes (280-510 minutes), which indicates the difficulties in cutting through postoperative adhesions. The loss of blood was estimated at 450 mL or 15.5% of the cases necessitated intraoperative transfusion. Clavien-Dindo Grade > III morbidity (major morbidity) was identified in 21.4% of the study population with the most frequent adverse events being intra-abdominal fluid collections and delayed gastric emptying. It showed a 90-day mortality rate of 2.4% that the procedure can be done with a satisfactory safety profile in high-volume special centers despite its complexity. The overall survival (OS) median of the entire cohort after undergoing the completion procedure was 32.5 months. The 5-year OS rate was computed to be 28.6, indicating a significant increase over historical palliative cohorts. Univariate analysis revealed multiple variables with a positive correlation with improved longevity, such as a DFI greater than 24 months (p < 0.001), pre-operative CA 19-9 level less than 37U/mL (p = 0.004) and the absence of lymph node metastasis in the second specimen (p = 0.012). A multivariate Cox proportional hazards model was created to determine independent predictors of 5-year survival. The analysis established that a DFI of over 24 months was a strong independent predictor of survival with Hazard Ratio (HR) of 0.42 (95% CI: 0.28- 0.64; p < 0.001).

## Discussion:

This study demonstrates that there is a lot of evidence that completion total pancreatectomy (CTP) is an effective therapy that can be used to help patients survive in the long term. Previously, it was considered that the recurrence in the remaining pancreas (IRRP) alone was an evidence of systemic failure and thus, surgeons tended to hesitate to perform additional surgery. Nevertheless, this negative perspective is not supported by our 5-year overall survival rate of 28.6% which suggests that localized failure can be an alternative, less aggressive form of pancreatic ductal adenocarcinoma (PDAC). This survival rate is equal to the recent multi-institutional studies and is much higher than the survival rate of palliative systemic chemotherapy or radiotherapy [10, 11]. Disease-free interval (DFI) was the most significant variable that we discovered during our analysis. DFI greater than 24 months was correlated with a large survival advantage indicating that period between initial surgery and recurrence is a biological filter. Late recurrence patients probably have a low growing tumor, whereas early recurrence (within 12 to 18 months) is a sign of undetectable systemic micro metastases that were not found during the initial operation. Thus, CTP having a short DFI is usually an ineffective treatment choice because the physical burden of complete pancreatectomy is rarely compensated by the negligible oncological advantage [9, 12]. In addition to the time aspect of DFI, preoperative serum CA 19-9 levels were also determined to be a good predictor of post-recurrence outcomes. High CA 19-9 levels are often associated with a greater risk of early systemic relapse after salvage surgery. A combination of CA 19-9 trends and DFI may help doctors to design a more precise biological profile of the patient so that CTP is administered only to those patients who are most likely to undergo a secondary R0 resection [3,13].

The complexity of CTP still remains a significant obstacle, since the operation requires navigating through dense retroperitoneal fibrosis and deformed vascular anatomy. Despite an equivalent rate of 21.4% of major complications, which is similar to that of the high-volume centers, the eventual transition to and a pancreatic state is a permanent morbidity. Pancreatogenic diabetes type 3c is a disease that can be extremely difficult to treat because of simultaneous loss of both insulin and glucagon, leaving patients prone to unstable glucose levels. However, our results indicate that a good quality of life can be achieved with the use of modern continuous glucose monitoring (CGM) and weight-adjusted enzyme replacement therapy that is sufficient to justify the further attention. There are limitations to this study related to being retrospective and selection bias inherent to surgical candidates. Moreover, we could not clearly differentiate between true late recurrence and metachronous primary tumors on the remnant.

## Conclusion:

Total Pancreatectomy (TP) is a viable and possibly curative therapy choice in a restricted number of patients, who experience isolated recurrence of PDAC in the remaining pancreatic tissue. Despite its doubtful reception in the past owing to the high probability of systemic occurrence, recent data show that in patients with good tumor features, the chances of systemic metastasis are low.

## Figures and Tables

**Table 1:** Clinicopathologic outcomes and survival predictors

**Variable**	**Total Cohort (N=84)**	**5-Year Survivors (n=24)**	**Non-Survivors (n=60)**	**p-value**
Median Age (Years)	66 (IQR 58-72)	64 (55-70)	68 (60-75)	0.142
Initial Operation				0.765
Pancreatoduodenectomy	52 (62%)	15 (62.5%)	37 (61.7%)	
Distal Pancreatectomy	32 (38%)	9 (37.5%)	23 (38.3%)	
Disease-Free Interval				< 0.001*
> 24Months	48 (57%)	20 (83.3%)	28 (46.7%)	
>24 Months	36 (43%)	4 (16.7%)	32 (53.3%)	
Pre-operative CA 19-9 (U/mL)				0.004*
< 37	54 (64%)	21 (87.5%)	33 (55%)	
> 37	30 (36%)	3 (12.5%)	27 (45%)	
Resection Margin				0.088
R0(Negative)	69 (82%)	23 (96%)	46 (77%)	
R1(Microscopic)	15 (18%)	1 (4%)	14 (23%)	
